# Frequency and predictors of unspecific medical diagnoses in the emergency department: a prospective observational study

**DOI:** 10.1186/s12873-022-00665-x

**Published:** 2022-06-15

**Authors:** Tanja Birrenbach, Michele Hoffmann, Stefanie C. Hautz, Juliane E. Kämmer, Aristomenis K. Exadaktylos, Thomas C. Sauter, Martin Müller, Wolf E. Hautz

**Affiliations:** 1 Department of Emergency Medicine, Inselspital, University Hospital, University of Bern, 3010 Bern, Switzerland; 2 Faculty of Medicine, Centre for Health Sciences Education, University of Oslo, Oslo, Norway

**Keywords:** Unspecific diagnoses, Non-specific complaints, Emergency department, Diagnostic error

## Abstract

**Background:**

Misdiagnosis is a major public health problem, causing increased morbidity and mortality. In the busy setting of an emergency department (ED) patients are diagnosed under difficult circumstances. As a consequence, the ED diagnosis at hospital admittance may often be a descriptive diagnosis, such as “decreased general condition”. Our objective was to determine in how far patients with such an unspecific ED diagnosis differ from patients with a specific ED diagnosis and whether they experience a worse outcome.

**Methods:**

We conducted a prospective observational study in Bern university hospital in Switzerland for all adult non-trauma patients admitted to any internal medicine ward from August 15th 2015 to December 7th 2015. Unspecific ED diagnoses were defined through the clinical classification software for ICD-10 by two outcome assessors. As outcome parameters, we assessed in-hospital mortality and length of hospital stay.

**Results:**

Six hundred eighty six consecutive patients were included. Unspecific diagnoses were identified in 100 (14.6%) of all consultations.

Patients receiving an unspecific diagnosis at ED discharge were significantly more often women (56.0% vs. 43.9%, *p* = 0.024), presented more often with a non-specific complaint (34% vs. 21%, *p* = 0.004), were less often demonstrating an abnormal heart rate (5.0% vs. 12.5%, *p* = 0.03), and less often on antibiotics (32.0% vs. 49.0%, *p* = 0.002). Apart from these, no studied drug intake, laboratory or clinical data including change in diagnosis was associated significantly with an unspecific diagnosis. Unspecific diagnoses were neither associated with in-hospital mortality in multivariable analysis (OR = 1.74, 95% CI: 0.60–5.04; *p* = 0.305) adjusted for relevant confounders nor with length of hospital stay (GMR = 0.87, 95% CI: 0.23–3.32; *p* = 0.840).

**Conclusions:**

Women and patients with non-specific presenting complaints and no abnormal heart rate are at risk of receiving unspecific ED diagnoses that do not allow for targeted treatment, discharge and prognosis. This study did not find an effect of such diagnoses on length of hospital stay nor in-hospital mortality.

**Supplementary Information:**

The online version contains supplementary material available at 10.1186/s12873-022-00665-x.

## Background

The practice of emergency medicine is considered “a natural laboratory for the study of error” [1]. In the emergency department (ED), patients are diagnosed under difficult circumstances, including high physician workload, medical urgencies, shift work, nonlinear workflow and overcrowding. All these factors impact medical decision making and cultural and language barriers may further impair communication with the patient. Moreover, particularly complex medical patients often present a diagnostic challenge. Still, it is the task of emergency physicians to assign a preliminary diagnosis to patients who are subsequently hospitalized.

From admission to discharge, the leading clinical diagnosis may change. Such change is often used as an indicator of diagnostic error in the ED [2]. Recently, a German university ED reported a change in diagnosis in 29% of their hospitalized patients [3]. Other studies estimated the rate of diagnostic errors at around 15–30% in contexts such as the ED [4–8]. About 45% of diagnostic errors result in moderate to severe patient harm [4, 9]. In a prospective observational study at a large Swiss university hospital ED, we recently demonstrated a change in diagnosis in ED patients admitted to the internal medicine ward to be associated with a longer hospital stay and substantially higher mortality [10].

One might argue that a change in lead diagnosis is a clinically relevant outcome parameter in itself: when a patient who initially requires hospital-admission improves to be discharged with an identical diagnosis, treatment was most likely adequate. When, however, a change in diagnosis occurs during hospitalization, the patient is at risk of missed, delayed or inadequate initial treatment. This may explain an increase in length of hospital stay, more unscheduled transfers to the intensive care unit, and even higher mortality. It further explains the many well-known effects of diagnostic error, such as medico-legal, economic, and social consequences [4, 11].

However, a change in diagnosis as an indicator of diagnostic error is only assessable in retrospect and not at the point of care. It thus does not help reduce or prevent patient harm. Here, we hypothesize that unspecific (“vague”) ED diagnoses, which include labels such as “generally degraded health status” or “fever of unknown origin”, may have similar effects on treatment and thus on subsequent outcome parameters, but are available in time to identify patients at risk. Unspecific ED diagnoses arguably have little therapeutic or prognostic value and potentially encompass a large variety of underlying diseases. Research into factors associated with such unspecific ED diagnosis and the outcome of patients receiving such unspecific diagnoses is scare, even though they may encompass up to 20–30% of ED patients [12–18].

This study thus aims to explore the following research questions:

1. What is the frequency of unspecific ED diagnoses among patients admitted to the internal medicine ward?
2. Do patients with unspecific ED diagnoses differ from patients with specific ED diagnoses in clinical aspects (e.g., age, gender, presenting complaint, severity of medical problem)?
3. Do patients with unspecific diagnoses experience a worse outcome than patients with specific diagnoses?

## Methods

### Study design, setting, and ethical approval

This is a secondary analysis of a dataset obtained during the cDx study, a prospective observational study following patients admitted to hospital through the ED [2]. This study found that patients who experienced a change in diagnosis from admittance to discharge had a significantly longer length of hospital stay and a significantly higher mortality than those who did not [10].

The study took place in the ED of the Bern University Hospital and included all non-trauma patients older than 18 years admitted to any internal medicine ward (IM) through the ED. The ED at Bern University hospital, a tertiary care center, is a self-contained, interdisciplinary unit that employs around 45 physicians and 120 nurses, and sees more than 45,000 patients per year, of which around 30% are admitted to the hospital [19]. The department of internal medicine cares for over 4,000 in-patient cases per year. The final ED diagnosis is made by the treating ED team, which consists of an attending physician (board certified in internal medicine and emergency medicine) and a resident. The patient left the ED with an ED diagnosis that is based on available previous medical records and the details of the actual presentation. Further details on study settings are reported in previous publications [2, 10, 20].

Patient data were collected during usual ED care and in the internal medicine ward. The Bern ethics committee registered the study as a quality evaluation study under Kantonale Ethikkomission Bern BASEC number 197/15 and waived the need for informed consent.

### Inclusion/Exclusion

All patients ≥ 18 years of age admitted to any internal medicine ward (IM) at Bern University Hospital via the ED during the five months study period were included.

**Fig. 1:**
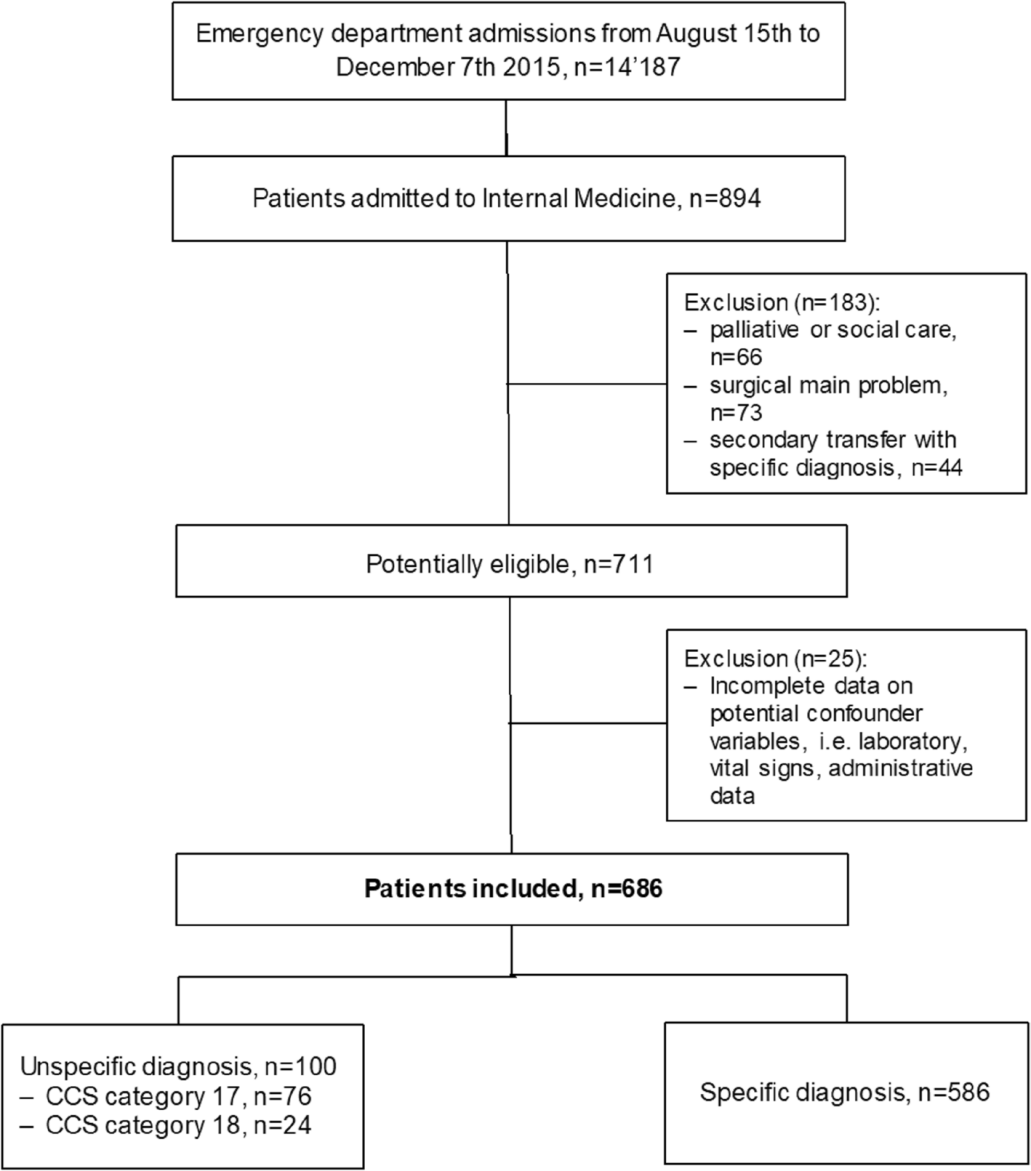
Flowchart

Exclusion criteria were patients admitted to the IM ward for palliative or social care as primary purpose, because diagnostic workup and treatment are different in this specific patient population. Also, patients with surgical main problems (e.g., fractures) admitted to the IM ward because of age or comorbidities were excluded for similar reasons. The third group of excluded patients were patients transferred to our tertiary hospital with a diagnosis established externally, because we assumed diagnostic workup in our ED not to be comparable to other patients. Furthermore, patients with incomplete information on the potential confounding variables were excluded. For an overview of the inclusion and exclusion procedure, see the study flowchart (Fig. 1).

### Data collection & extraction

The following data were collected from the hospital’s electronic patient documentation systems (E-Care, ED 2.1.3.0, Turnhout Belgium and i-pdos_Prod_ODA 7.10.1.2): *patient characteristics* (date of birth, sex, date and time of presentation, prescribed medication, insurance status), *encounter characteristics* (Swiss triage scale, ED admittance via resuscitation bay, first measured blood pressure and heart rate, the total diagnostic resources), *consultation characteristics* (creatinine, sodium, hemoglobin, leucocytes, hemoglobin) and *outcome data*(length of stay in the ED and hospital, discharge diagnoses from the ED and IM, and in-hospital mortality). All patients admitted to the ED were triaged by registered nurses using the Swiss triage scale, a five-level triage scale with high inter-rater and intra-rater reliability. Chief complaints, objective parameters (vital signs), and key questions are used to stratify the risk: life-threatening emergencies requiring immediate care, urgent conditions requiring medical evaluation within 20 min, semiurgent conditions, requiring medical evaluation within 2 h, nonurgent conditions and follow-ups [21].

### Diagnoses according to the ICD-10 classification

Patient records were reviewed to establish the corresponding final diagnosis according to the 10^th^ International Classification of Diseases and Health Related Problems (ICD-10) [22] by two outcome assessors, experienced in internal and emergency medicine. In cases where a specific organ-related ICD-diagnosis could not be established, the presenting clinical symptom was coded (e.g., “decreased general condition” was attributed to R53). Because ED diagnoses are often descriptive, specific coding rules were established (supporting information S1 Table). For example, sepsis was coded under the corresponding infection (e.g., “sepsis due to pneumonia” was coded as “pneumonia”).

One hundred randomly selected patients were independently classified by both raters to assess their rater agreement (kappa = 0.96). The remaining cases were then classified by one rater.

ICD codes were grouped into diagnostic categories taken from the clinical classification software (CCS) for ICD-10 [23]. The software groups diseases by ICD 10 code in 18 different groups such as “cardio-circulatory diseases” or “infectious diseases”.

### Classification of diagnoses

All diagnoses classified as “residual codes” or “symptoms; signs, ill-defined conditions and factors influencing health status” by the CCS (categories 17 and 18) were summarized as *unspecific diagnoses,* including labels such as “generally degraded health status” or “fever of unknown origin.”

### Outcomes

The primary outcome was in-hospital mortality. Secondary outcome was length of stay in hospital.

### Potential confounders

The following variables were considered as potential confounders:

i. *Sociodemographic parameter* including age, gender and insurance status
ii. *Drug intake*, i.e., antihypertensive, diuretic, antidiabetic, antiepileptic, psycholeptic, antibiotic, or antithrombotic therapy
iii. *Acute consultation characteristics*, i.e., triage scale, resuscitation bay use, heart rate deviation (below 50 bpm or above 110 bpm) and reduced blood pressure below 90 mmHg [24].
iv. *Additional laboratory characteristics known to be associated with poor outcome*(sodium, creatinine, hemoglobin, and leucocyte count) [25].
v. *Diagnostic ED resources* measured in tax points as the sum of radiological, laboratory and physician’s work. Each service is assigned a certain number of tax points depending on the time required, the difficulty and the infrastructure required. This is used for standardization for billing/accounting reasons in the Swiss healthcare system. 1 tax point roughly is the equivalent to 1 Swiss Franc, varying from time and region.
vi. Specificity of the presenting complaint: presenting complaints, for which no evidence-based management protocols for the ED exist [26], were coded by two independent raters as described previously as unspecific, so called non-specific complaint (NSC) [27], or as specific otherwise.

### Sample size motivation

The in-hospital mortality in our cohort in the specific diagnosis group and the proportion of unspecific diagnoses of patients admitted to the hospital ward from the ED were estimated to be 4.3% and 21% respectively[10] (in analogy to Wogan et al. studying hospital admissions from the ED) [15]. Furthermore, we assumed a strong impact of an unspecific diagnosis because of the strong impact of a discrepancy between the ED and IM ward discharge diagnosis, which was found to result in an OR of 2.4 [10]. Assuming these numbers, 644 consultations would be sufficient to detect an OR for mortality of 2.4 between specific vs. unspecific ED discharge diagnosis with a power of 70% and a significance level of 0.05.

### Statistical analysis

Data was analysed in Stata® 16.1 (StataCorp, The College Station, Texas, USA).

Baseline characteristics are presented as numbers and percentage or median and interquartile range (IQR) using descriptive statistics as appropriate. Groups of patients with unspecific and specific diagnoses are compared regarding presentation, ED and hospital outcome with Mann–Whitney U tests or Chi-square tests as applicable.

A stepwise backward logistic (respectively linear regression) analysis (maintaining all predictors with a *p* < 0.1) based on all potential confounders described above was used to control the association of an unspecific diagnosis and in-hospital mortality (or length of hospital stay) for confounding using Stata’s – stepwise – command.

The effect sizes of the logistic regression are presented as odds ratio and 95% confidence interval (CI). As length of hospital stay (los) was not normally distributed, it was ln-transformed. The coefficients and 95% CI of the linear regression model to predict ln-los were exponentiated, thus, corresponding to the geometric mean ratio (GMR) accompanied by the 95% CI.

## Results

A total of 14,187 non-trauma medical patients presented to the ED during the study period, of which 894 patients were admitted to an IM ward (Fig. 1). From those, 183 (20.4%) were excluded because they met our exclusion criteria, 25 (3.5%) had incomplete data on any of the studied confounder and were therefore excluded too, leaving 686 consultations for analysis.

In total, 100 (14.6%) of the 686 included patients received an unspecific diagnosis in the ED at hospital admission, of which 76 (11.1% of all) were classified in CCS category 17 (“symptoms; signs; and ill-defined conditions and factors influencing health status”) and the remainder 24 (3.5% of all) in CCS categories 18 (“residual codes”).

Table 1 summarizes the ED diagnoses patients received at hospital admittance by their respective CCS category. The most common specific categories were diseases of the circulatory (18.2%), respiratory (15.6%), and digestive system (12.1%).

**Table 1:** Summary of diagnoses patients received in the emergency department, according to the clinical classification system (CCS)

CCS Level 1 category	Freq. (n)	Relative freq. (%)
**Specific diagnoses**		
Diseases of the circulatory system	125	18.2
Diseases of the respiratory system	107	15.6
Diseases of the digestive system	83	12.1
Endocrine; nutritional; and metabolic diseases and immunity disorders	43	6.3
Diseases of the nervous system and sense organs	40	5.8
Diseases of the genitourinary system	37	5.4
Infectious and parasitic diseases	33	4.8
Injury and poisoning	28	4.1
Mental illness	28	4.1
Diseases of the musculoskeletal system and connective tissue	27	3.9
Neoplasms	17	2.5
Diseases of the blood and blood-forming organs	10	1.5
Diseases of the skin and subcutaneous tissue	7	1
Congenital anomalies	1	0.2
**Total specific diagnoses**	**586**	**85.4**
**Unspecific diagnoses**		
Symptoms; signs; and ill-defined conditions and factors influencing health status	76	11.1
Residual codes; unclassified	24	3.5
**Total unspecific diagnoses**	**100**	**14.6**
**Total overall**	**686**	**100.0**

**Fig. 2:**
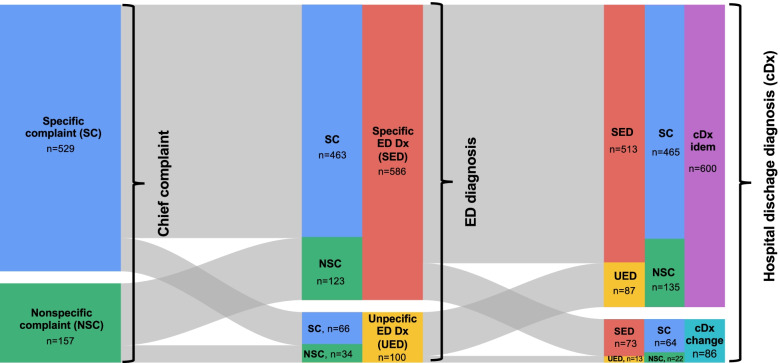
Relation between presenting chief complaint, ED diagnosis, and hospital discharge diagnosis. Abbreviations: cDx, Hospital Discharge Diagnosis; ED, Emergency Department; NSC, Non-Specific Complaint; SC, specific complaint; SED, Specific ED Diagnosis; UED, Unspecific ED Diagnosis

The relation/the flow between presenting chief complaint (specific vs. nonspecific), ED diagnosis (specific vs. unspecific) and hospital discharge diagnosis (change in diagnosis vs. idem diagnosis as ED diagnosis) is demonstrated in Fig. 2.

Table 2 provides a comparison of patients receiving unspecific vs. specific diagnoses. Patients receiving an unspecific diagnosis at ED discharge were significantly more often women (56.0% vs. 43.9%, *p* = 0.024), presented more often with a NSC (34% vs. 21%, *p* = 0.004), were less often demonstrating a heart rate deviation (5.0% vs. 12.5%, *p* = 0.03), and less often on antibiotics on admission to the IM ward (32.0% vs. 49.0%, *p* = 0.002). Furthermore, their median length of stay in the ED was longer (6.75 vs. 6.2 h, *p* = 0.036). Apart from these, no studied drug intake, laboratory or clinical data was significantly associated with an unspecific diagnosis.

**Table 2:** Comparison of patients with unspecific versus specific ED diagnoses

	**Unspecific diagnosis (*n* = 100)**		**Specific diagnosis (*n* = 586)**		***p*-value**
**At admittance**					
Age [median (IQR)]	68.5	(55.5–77)	70.0	(54–80)	0.361
Gender [n (%) female]	56	(56)	257	(43.9)	0.024
Private insurance, [n (%)]	21	(21)	109	(18.6)	0.572
NSC, [n (%)]	34	(34)	123	(21)	0.004
Time of admission, [n (%)]					0.300
Day (6 am – 5 pm)	58	(58)	342	(58.4)	
Evening (5 pm – 10 pm)	32	(32)	156	(26.6)	
Night (10 pm – 6 am)	10	(10)	88	(15)	
**Medication, [n (%)]**					
On antihypertensive therapy	61	(61.0)	322	(54.9)	0.260
On diuretic therapy	35	(35.0)	229	(39.1)	0.438
On antidiabetic therapy	15	(15.0)	63	(10.8)	0.216
On antiepileptic therapy	12	(12.0)	83	(14.2)	0.563
On psycholeptic therapy	45	(45.0)	309	(52.7)	0.153
On antibiotic therapy	32	(32.0)	287	(49.0)	0.002
On antithrombotic therapy	73	(73.0)	444	(75.8)	0.553
**In the ED**					
Triage category [n (%)]					0.291
Life-threatening	3	(3.0)	34	(5.8)	
Urgent conditions	39	(39.0)	247	(42.2)	
Semiurgent conditions	57	(57.0)	282	(48.1)	
Nonurgent conditions	1	(1.0)	19	(3.2)	
Follow-up	0	(0.0)	4	(0.7)	
Resuscitation bay, [n (%)]	5	(5.0)	66	(11.3)	0.057
Heart rate deviation, > 110/ < 50 bpm, [n (%)]	5	(5.0)	73	(12.5)	0.030
Blood pressure deviation, < 90 mmHg, [n (%)]	2	(2.0)	19	(3.2)	0.505
**Laboratory findings, [median (IQR)]**					
Sodium, [mmol/L]	137.0	(133–139)	137.0	(134–140)	0.153
Creatinine, [µmol/L]	81.0	(64.5–103)	79.0	(65–103)	0.663
Hemoglobin, [g/L]	126.0	(110.5–137)	126.5	(108–139)	0.920
Leucocyte count, [G/L]	8.8	(6.3–10.9)	8.9	(6.5–11.8)	0.594
Diagnostic ED resources, ln-transformed [in tax points; median (IQR)]	7.1	(6.6–7.5)	7.1	(6.8–7.4)	0.574
ED LOS [in hours; median (IQR)]	6.75	(5.4–8.3)	6.2	(4.6–7.8)	0.036
**Outcome**					
In-hospital mortality, [n (%)]	5	(5.0)	29	(4.9)	0.983
Change in diagnosis, [n (%)]	13	(13.0)	73	(12.5)	0.880
Hospital LOS [in days; median (IQR)]	4.8	(3–8.6)	5.7	(3.6–9.1)	0.141

*ED* Emergency Department, *IQR* Interquartile Range, *LOS* Length of Stay, *NSC* Non-Specific Complaint

Unspecific diagnoses were not associated with in-hospital death in multivariable analysis (OR = 1.74, 95% CI: 0.60–5.04; *p* = 0.305), adjusted for age, medical intake (antihypertensive, antithrombotic, and psycholeptic therapy), need for resuscitation bay, blood pressure deviation (< 90 mmHg) as well as hemoglobin level (Table 3). Length of hospital stay was not significantly associated with unspecific diagnoses in backward regression analysis (GMR = 0.87, 95% CI: 0.23–3.32; *p* = 0.840).

**Table 3:** Multivariable analysis; stepwise backward logistic regression

In-hospital-mortality	Odds ratio	95% Confidence interval	*p*-value
**Unspecific diagnosis, [yes]**	1.74	(0.60—5.04)	0.305
**At admittance**			
Age, [per year older]	1.1	(1.02—1.08)	< 0.001
**Medication**			
On antihypertensive therapy, [yes]	0.3	(0.13—0.65)	0.003
On antithrombotic therapy, [yes]	0.4	(0.18—1)	0.050
On psycholeptic therapy, [yes]	2.4	(1.05 – 5.31)	0.037
**In the ED**			
Resuscitation bay, [yes]	5.1	(2.11—12.28)	< 0.001
Blood pressure deviation, < 90 mmHg, [yes]	7.1	(2.11—24.22)	0.002
Hemoglobin, [per g/L]	1.0	(0.97—1)	0.01

*ED* Emergency Department

## Discussion

Every seventh patients (14.6%) left the ED for the IM ward with an unspecific ED diagnosis. Females and patients presenting with a NSC were more likely to receive an unspecific diagnosis. In contrast, consultations with an abnormal heart rate (< 50 or > 110 bpm) and patients prescribed an antibiotic therapy in hospital had lower odds for an unspecific diagnosis.

Our estimate of the frequency of unspecific ED diagnosis is at the lower end of the scale of published estimates. A nationally representative study in the United States looking at diagnoses of patients presenting with the three most common ED chief complaints, chest pain, abdominal pain, and headache in ambulatory patients found that 37% of patients were discharged from the ED with a symptomatic instead of a specific pathological diagnosis [12]. Similarly, unspecific diagnoses (“signs and symptoms”) accounted for over one-third of ED discharge diagnoses of outpatients in a single academic center [28], and over 20% in an Icelandic investigation [17]. In a large Danish cohort study of patients brought to the hospital in an ambulance dispatched after emergency calls, unspecific diagnoses accounted for one-third of patients [14]. Analyzing an US American sample, Wogan has pointed out that 23% of patients admitted to a hospital ward form the ED did not have a causative diagnosis [15]. In elderly patients in France, this proportion even reaches one third [16].

A reason for the difference between these and our estimate may be that several countries limit the time spent in the ED to a maximum, e.g. 4 h. Patients may afterwards be transferred to a medical investigation unit for another maximum of e.g. 24 h before finally deciding on admission. Our ED, like many other European EDs, combines these two functions within a single unit, and allows for certain patients to stay in the ED until the diagnostic/therapeutic process has advanced (median in our sample: approx. 7 h) possibly yielding/ allowing for a more definite/specific diagnosis. This may explain the comparatively lower rate of unspecific ED diagnoses found here. Furthermore, the effect of a tertiary care center with the corresponding diagnostic possibilities might have had an influence on the lower rate of unspecific ED diagnoses.

Patients presenting with a NSC were more likely to receive an unspecific diagnosis in our study. Several studies have found an increased mortality in patients with NSC [29–32]. Indeed, patients presenting to the ED with NSC present a challenge to the treating physician, and are at an increased risk of misdiagnosis, admission to hospital, prolonged stay in hospital and even mortality [27, 29–33]. It has thus been hypothesized, that a low quality ED diagnosis in this patient group may be a factor contributing to impaired outcome. Our results do not suggest so. Patients with an unspecific diagnosis at ED discharge were not found to have an increased risk of in-hospital mortality in multivariable analyses adjusted for relevant confounders nor an increased length of stay. It thus remains an open question for further research, how non-specific complaints worsen outcome of patients hospitalized [27, 30, 32, 34] if they do not do so through the quality of ED diagnoses.

In a previous study, we found a change in diagnosis in ED patients admitted to the internal medicine ward in 12.3% of patients, associated with a longer hospital stay and higher mortality [10]. We initially hypothesized that an unspecific ED discharge diagnosis should be associated with a change in diagnosis when comparing ED and hospital discharge diagnoses. Surprisingly, this was not the case: The rate of change in diagnosis in this study was not significantly different in patients with a specific diagnosis vs. unspecific diagnosis (12.5% vs. 13%, *p* = 0.880; Table 2). Instead, we found that just as NSC do not necessarily lead to an unspecific ED diagnosis, only a small portion of those with an unspecific ED diagnosis experience a change in their final diagnosis when discharged from the hospital (Fig. 2). This finding implies that at least some patients admitted to the hospital with an unspecific diagnosis from the ED are discharged from the hospital with no improvement in the specificity of their diagnosis, a finding that we did not expect.

We do not know the reason for that lack of improvement in diagnosis specificity. One could hypothesise that when patients admitted to the hospital from the ED improve in their health over the next few days, hospitalists have little reason to revisit (and potentially change) the initial ED diagnosis. Maybe such a change in diagnosis is only triggered by a lack of patient improvement on the ward.

Next to unspecific presenting complaints, female gender was associated with receiving an unspecific diagnosis. Similarly, in patients presenting with NSC to another Swiss ED, diagnoses were more often missed in women [33], a phenomenon also known for acute coronary syndrome [35], and psychiatric diseases [36]. This may be attributable to a less specific presentation of some diseases in female patients (e.g. urinary tract infection, coronary artery disease), or even due to a bias towards psychogenic explanations for women [35].

Some authors conclude older patients to be more difficult to diagnose, perhaps because some symptoms may be underestimated or clinically less pronounced, attributed to aging in general, or because some of the diagnostic criteria might not be applicable to the elderly [4, 6]. Interestingly, in this study, no association between age and an unspecific ED diagnosis could be detected.

As a last finding, diagnostic resource consumption in the ED was not statistically different in patients receiving an unspecific versus a specific diagnosis. This finding implies a comparable diagnostic effort invested in both groups, which is somewhat surprising, given that ED physicians tend to order more diagnostic tests in cases about which they are uncertain [37]. The finding that physicians did not invest more diagnostic effort in patients eventually receiving a vague diagnosis calls the calibration of their confidence to actual diagnostic performance into question [38].

Sir William Osler stated that “Medicine is a science of uncertainty, and an art of probability” [39]. The uncertainty inherent in medical diagnosis is easily masked by disease labels [40]. It may be worthwhile to actually communicate our level of certainty in our diagnosis [40], as case-related confidence has previously been shown to be associated with the actual accuracy of medical diagnoses [41–44]. Our findings raise the question whether this association holds for patients with non-specific complaints and those receiving unspecific ED diagnoses eventually.

### Limitations

Our study is limited to a single university center, albeit one of the largest of its kind in Switzerland. Thus, the generalizability of our findings to other populations and other ED settings remains unknown. Because we only included patients hospitalized to IM, we cannot rule out that our inclusion criteria introduced selection bias. This study did not include patients with a non-specific surgical problems. However, these problems are rare, because most presenting complaints are only identified as surgical if they can be diagnosed as a specifically addressable surgical problem. The CI for some of the analyses was rather wide, this might imply that the population is too small to make a more precise estimate. Furthermore, the sample size was small, thus the power might not have been enough to detect a smaller strength of association between non-specific diagnosis and in-hospital mortality than calculated. We also cannot expand our findings to the outpatient-population. Furthermore, we did not include information on 4-week mortality, but only on in-hospital mortality.

## Conclusion

In this prospective, observational study, an unspecific ED diagnosis was more often made in females and patients presenting with a NSC. Consultations with an abnormal heart rate and patients on antibiotics had lower odds for an unspecific diagnosis. Unspecific ED diagnoses were not associated with impaired patient outcomes. Thus, unspecific diagnoses seem not be the primary factor driving in-house mortality and length of stay in patients presenting with NSC. More research is needed to uncover the complex relation between NSC, unspecific diagnoses and patient outcomes.

## Supplementary Information

**Additional file 1: S1 Table.** Specific ICD-10 coding rules.

## Data Availability

Data contain potentially identifying or sensitive patient information. Data used in this study are available upon reasonable request from the corresponding author at the Emergency Department of the University Hospital Bern, Switzerland to researchers eligible under Swiss legislation to work with codified personal health care data. Eligibility will be determined by Cantonal ethics committee Bern.

## Abbreviations

BPM: Beats Per Minute
CCS: Clinical Classification Software for ICD-10
CI: Confidence Interval
ED: Emergency Department
GMR: Geometric Mean Ratio
ICD-10: 10^th^ International Classification of Diseases and Health Related Problems
IM: Internal Medicine
IQR: Interquartile Range
LOS: Length of Stay
NSC: Non-Specific Complaint
OR: Odds Ratio
