# Supplementary material for: Frequency and predictors of unspecific medical diagnoses in the emergency department: a prospective observational study
Source: BMC Emerg Med. 2022 Jun 15;22:109. doi: 10.1186/s12873-022-00665-x (PMC9199121; doi:10.1186/s12873-022-00665-x)
Supplement: Supplementary file 1 — Additional file 1: S1 Table. Specific ICD-10 coding rules. [file 12873_2022_665_MOESM1_ESM.docx]

**S1 Table. Specific ICD-10 coding rules**

| **Rule number** | **Definition** | **Example** |
| --- | --- | --- |
| 1 | Specific diagnosis available in discharge letter | Discharge diagnosis “bronchial asthma” will be coded as “bronchial asthma J45.9” |
| 2 | If specific organ-related ICD-diagnosis cannot be established, the presenting clinical symptom is to be coded | Discharge diagnosis “decreased general condition” will be coded as “R53” |
| 3 | Suspected diagnosis will be coded as the definitive diagnosis | Discharge diagnosis “suspected bronchial asthma” will be coded as “bronchial asthma J45.9” |
| 4 | Nonspecific symptoms and ONE specific differential diagnosis or ONE suspected specific diagnosis will be coded as the specific diagnosis | Discharge diagnosis “Dyspnoe, suspected bronchial asthma” will be coded as “bronchial asthma J45.9”  Discharge diagnosis “Dyspnoe, differential diagnosis bronchial asthma” will be coded as “bronchial asthma J45.9” |
| 5 | Nonspecific symptoms and several differential diagnoses will be coded as nonspecific symptoms | Discharge diagnosis “Dyspnoe, differential diagnosis bronchial asthma, hyperventilation, pneumonia” will be coded as “Dyspnoe R06” |
| 6 | Specific diagnosis and ONE differential diagnosis will be coded as the specific diagnosis | Discharge diagnosis “bronchial asthma, differential diagnosis pneumonia” will be coded as “bronchial asthma J45.9” |
| 7 | Specific diagnosis and more than one differential diagnoses will be coded as the specific diagnosis | Discharge diagnosis “bronchial asthma, differential diagnosis pneumonia, hyperventilation, pulmonary embolism” will be coded as “bronchial asthma J45.9” |
| 8 | If discharge diagnosis consists of a neoplasm with a current problem, the current problem will be coded | Discharge diagnosis “non-small cell lung cancer, current problem pneumonia” will be coded as “pneumonia J18.9” |
| 9 | If discharge diagnosis consists of a neoplasm without a current problem, the neoplasm will be coded | Discharge diagnosis “metastatic breast cancer, initially pT2 pN2 M1” will be coded as “breast cancer C50” |
| 10 | Sepsis will be coded under the corresponding infection | Discharge diagnosis “Sepsis due to pneumonia” will be coded as “pneumonia J18.9” |
| 11 | No coding possible | Discharge diagnosis “xxx” cannot be coded |
